# Fabrication of porous nanoflake BiMO_*x*_ (M = W, V, and Mo) photoanodes *via* hydrothermal anion exchange†
†Electronic supplementary information (ESI) available: Experimental SEM images and the amount of hydrogen evolution of Bi_2_WO_6_, BiVO_4_, and Bi_2_MoO_6_ photoelectrodes. See DOI: 10.1039/c6sc01803c



**DOI:** 10.1039/c6sc01803c

**Published:** 2016-06-24

**Authors:** Jijie Zhang, Tuo Wang, Xiaoxia Chang, Ang Li, Jinlong Gong

**Affiliations:** a Key Laboratory for Green Chemical Technology of Ministry of Education , School of Chemical Engineering and Technology , Tianjin University , Collaborative Innovation Center of Chemical Science and Engineering , Tianjin 300072 , China . Email: jlgong@tju.edu.cn ; Fax: +86 22 87401818

## Abstract

Most Bi-based photoelectrodes have suitable band gaps and can effectively promote the water oxidation reaction. However, simple preparation methods for Bi-based binary metal oxides as photoanodes are scarce. This paper describes a simple hydrothermal anion exchange method to synthesize Bi-based binary metal oxides with controlled morphologies. This synthesis process uses BiOI as the template and Bi source, which is eventually converted to Bi-based porous nanoflake photoanodes upon reaction with MO_*x*_ (M = W, V, and Mo)-containing precursors. The photoanodes show well-shaped porous nanoflake morphologies and exhibit impressive photoelectrochemical properties compared to Bi-based photoanodes synthesized by conventional methods. These three samples possess long-term stability under solar irradiation and show considerable photocurrent for sulfite oxidation.

## Abstract 2

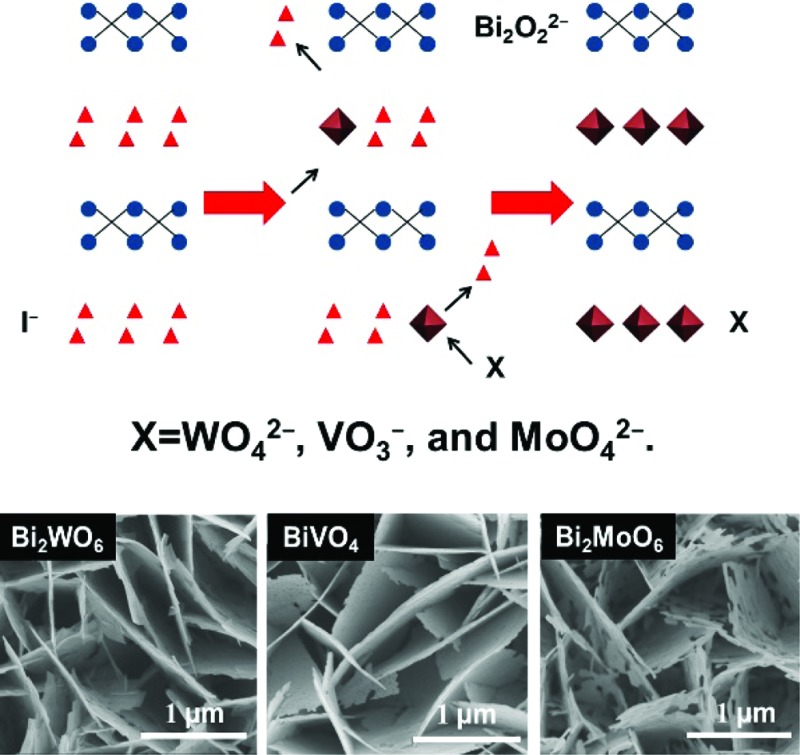
This paper describes a simple hydrothermal anion exchange method to synthesize Bi-based binary metal oxides (BiMO*_x_* (M = W, V, and Mo)) with controlled morphologies used as photoanodes for photoelectrochemical water oxidation.

## 1. Introduction

Photoelectrochemical (PEC) water splitting into hydrogen, driven by visible light, has attracted worldwide attention as a promising method to use solar energy.^1–4^ In the aspect of photoelectrode preparation, there are many requirements, such as a simple process and better controllability. Specifically, obtaining a large specific area and good crystallinity is sometimes a major concern in choosing the preparation method. For example, TiO_2_ photoelectrodes formed by various preparation methods exhibit different PEC performances. Zhang *et al.* synthesized TiO_2_ thin film electrodes using a sol–gel method and the maximum photocurrent value was just 0.5 μA cm^–2^.^5^ Another *in situ* growth method that formed branched TiO_2_ nanorods on fluorine-doped tin oxide (FTO) led to a much higher photocurrent of 1.1 mA cm^–2^ at 0.0 V *vs.* Ag/AgCl.^6^ The improved photocurrent was ascribed to the fine crystallinity and increased surface area due to the better transportation of charge carriers and more reactive sites.

Compared with single metal oxides, very few simple and effective preparation methods of binary metal oxides have been developed. In the past decades, binary metal oxides containing Bi(iii) have been identified as promising semiconductor electrodes in solar energy conversion.^7–9^ BiMO_*x*_ (M = V, C, P, W and Mo) has been widely applied in photodegradation and photoelectrochemical reactions.^10–14^ Among these popular Bi-based semiconductors, Bi_2_WO_6_, BiVO_4_, and Bi_2_MoO_6_ can absorb visible light and have suitable band gaps to drive the water oxidation reaction to generate H_2_ and reduce CO_2_ to form other useful fuels.^15^ Compared to the benchmark TiO_2_ which only responds to UV light, most Bi-based binary metal oxides can drive the water oxidation reaction under visible light irradiation.^8,16^ The common preparation methods for Bi-based binary metal oxides are dip-coating and spin-coating. However, the photoelectrodes synthesized by these conventional preparation methods show a planar structure and have a smaller specific area.^17,18^ Developing new preparation methods for BiMO_*x*_ photoanodes is an effective approach to enhance the photoactivity. For example, Bi_2_WO_6_ (2.7–2.8 eV) is a photocatalyst for PEC water oxidation and photodegradation under visible light irradiation.^19,20^ Generally, Bi_2_WO_6_ thin films are synthesized by directly coating Bi_2_WO_6_ nanoparticles on an electrode substrate using spin-coating,^21^ dip-coating,^22^ or electrostatic self-assembly deposition.^23^ However, these existing coating techniques often result in compact films with relatively low activities due to the small specific area.^24^ Only limited success has been reported for the direct synthesis of Bi_2_WO_6_ films. Recently, Zhang *et al.* developed a hard-template-directed sol–gel method to prepare porous Bi_2_WO_6_ thin films.^25^ Subsequently, they synthesized a Bi_2_WO_6_ photonic crystal film for visible-light-driven activity using a similar method.^26^ They synthesized Bi_2_WO_6_ photoelectrodes with a large specific area to enhance the light absorption. Most recently, Choi and co-workers prepared nanoporous BiVO_4_ electrodes by first annealing BiOI and a vanadium precursor in air.^27^ They created the BiVO_4_ porous photoanodes by directly forming the sample on the substrate to enhance the crystallinity. We believe that if Bi-based electrodes have both ordered morphology and good crystallinity, their PEC performances will be further improved.

This paper describes an effective hydrothermal anion exchange route based on BiOI substrates to fabricate porous nanoflake electrodes of Bi_2_WO_6_ (details in ESI†). This preparation method is unique in the sense that it can directly form the electrodes on the substrate, *e.g.*, *via* an *in situ* growth process, and results in Bi_2_WO_6_ electrodes with ordered morphology. To verify the universality in dendritic Bi-based electrodes, we also prepared BiVO_4_ (∼2.4 eV) and γ-type Bi_2_MoO_6_ (∼2.5 eV) porous nanoflakes with this hydrothermal anion exchange route.

## 2. Results and discussion

### 2.1 Crystal structure, morphology, and optical characterization of Bi_2_WO_6_, BiVO_4_, and Bi_2_MoO_6_

**Scheme 1:**
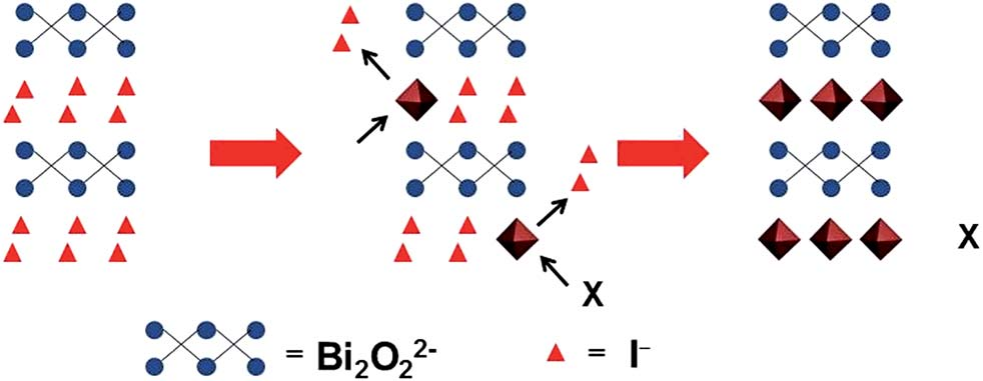
Schematic illustration of the hydrothermal anion exchange: from BiOI to Bi-based binary metal oxide. X = WO_4_^2–^, VO_3_^–^ or MoO_4_^2–^.

Anion exchange was originally used for forming photocatalyst nanoparticles with a core–shell structure. Alivisatos *et al.* used an anion exchange process to form single-crystalline hollow ZnS nanoparticles.^28^ Unlike cation exchange, the anion exchange in this work is accompanied by the nanoscale Kirkendall effect, yielding hollow nanoparticles. Meanwhile, it was thought that the anion exchange method could be extended to other binary or tertiary nanoparticles to produce hollow nanostructures. Then, Huang *et al.* synthesized Bi_2_WO_6_ hollow microspheres with a similar process, and used the Bi_2_WO_6_ microspheres to adsorb and reduce CO_2_ to methanol under visible light irradiation.^29^ In this paper, we extend this method to synthesize Bi-based photoanodes. First, the BiOI nanoflake arrays are electrodeposited on a FTO substrate. Then, the I^–^ ions are replaced by WO_4_^2–^ during a hydrothermal process. Finally, the Bi_2_WO_6_ electrodes are synthesized after annealing. Additionally, WO_4_^2–^ can be replaced by VO_3_^–^ or MoO_4_^2–^ to synthesize BiVO_4_ and Bi_2_MoO_6_ *via* this hydrothermal anion exchange reaction, which is a general and effective process for preparing Bi-based photoanodes with nanoflake morphology. The reaction mechanism of the anion exchange is shown in Scheme 1.

**Fig. 1:**
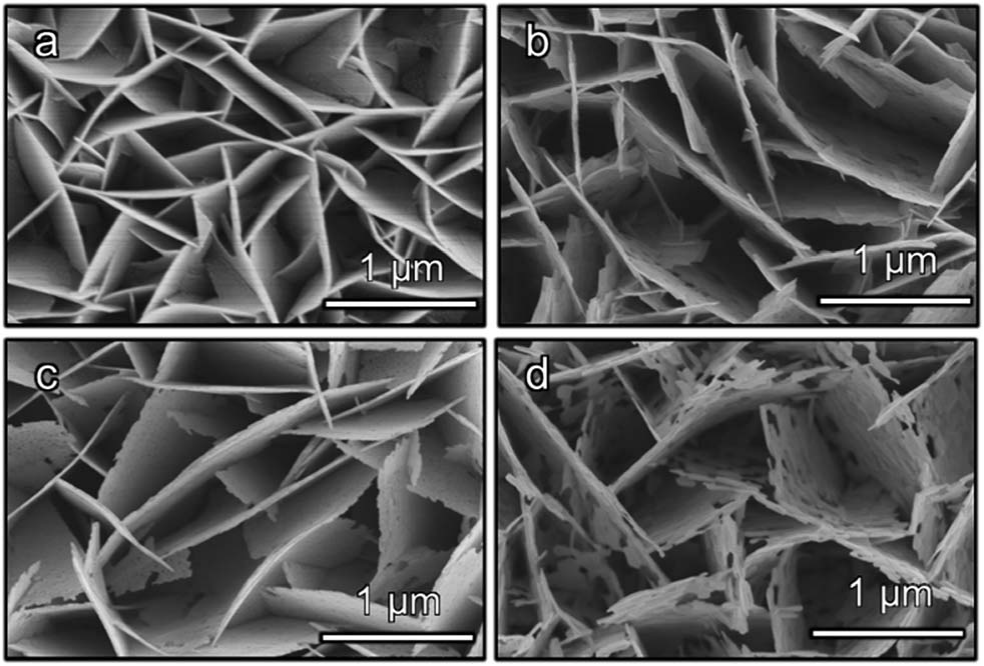
FESEM images of (a) BiOI, (b) Bi_2_WO_6_, (c) BiVO_4_, and (d) Bi_2_MoO_6_.

Field-emission scanning electron microscopy (FESEM) images reveal that the three Bi-based binary metal oxides have a flake-like nanostructure (Fig. 1). As the starting templates, the BiOI films show a nanoflake morphology (Fig. 1a) with a 2 μm height (Fig. 1a and S1a†). After the following hydrothermal anion exchange, the Bi-based binary metal oxides show irregular porous nanostructures (Fig. 1b–d), while the height is retained and is the same as the BiOI template (Fig. S1b–d†). The specific surface areas of the three Bi-based photoelectrodes (Bi_2_WO_6_, BiVO_4_ and Bi_2_MoO_6_) are about 13.4 m^2^ g^–1^, 27.3 m^2^ g^–1^ and 30.0 m^2^ g^–1^ respectively. The specific surface areas were calculated from the isotherms using the Brunauer–Emmett–Teller (BET) method and P-25 nanoparticles were used as the internal standard (details in ESI†).

**Fig. 2:**
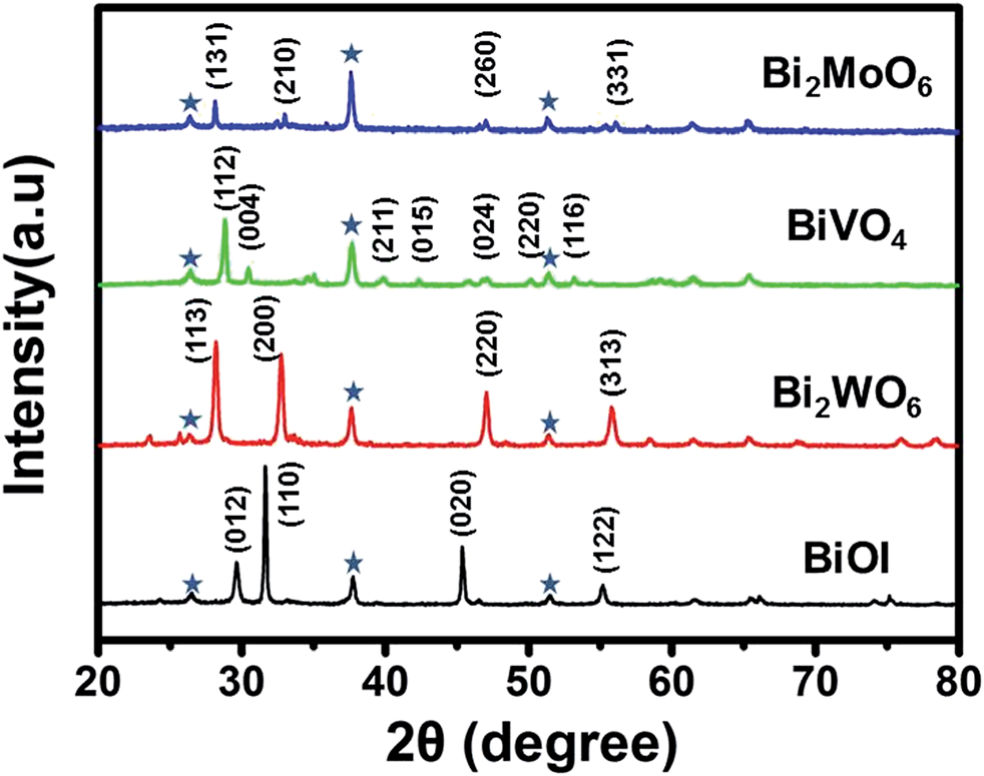
XRD patterns of BiOI, Bi_2_WO_6_, BiVO_4_ and Bi_2_MoO_6_. Peaks marked with an asterisk originate from the FTO substrate.

The crystallinity of the resulting Bi_2_WO_6_, BiVO_4_, and Bi_2_MoO_6_ electrodes is shown in the X-ray diffraction (XRD) patterns (Fig. 2). All of the three samples are well crystallized, and are consistent with orthorhombic Bi_2_WO_6_ (JCPDF 39-0256), monoclinic BiVO_4_ (JCPDF 83-1699), and orthorhombic Bi_2_MoO_6_ (JCPDF 77-1246). The atomic Bi : W, Bi : V, and Bi : Mo ratios in the Bi_2_WO_6_, BiVO_4_, and Bi_2_MoO_6_ electrodes were confirmed to be the expected atomic metal ratios (ESI, Table S1†) by energy-dispersive X-ray spectroscopy (EDS). Fig. S2† shows transmission electron microscopy images of the three samples. The high-resolution transmission electron microscopy (HRTEM) images indicate that the fringe spacing values are 0.2735 nm, 0.3089 nm and 0.3125 nm, which are consistent with the (200) plane of Bi_2_WO_6_, the (112) plane of BiVO_4_, and the (131) plane of Bi_2_MoO_6_, respectively. This result is in accordance with the XRD patterns (Fig. 2).

**Fig. 3:**
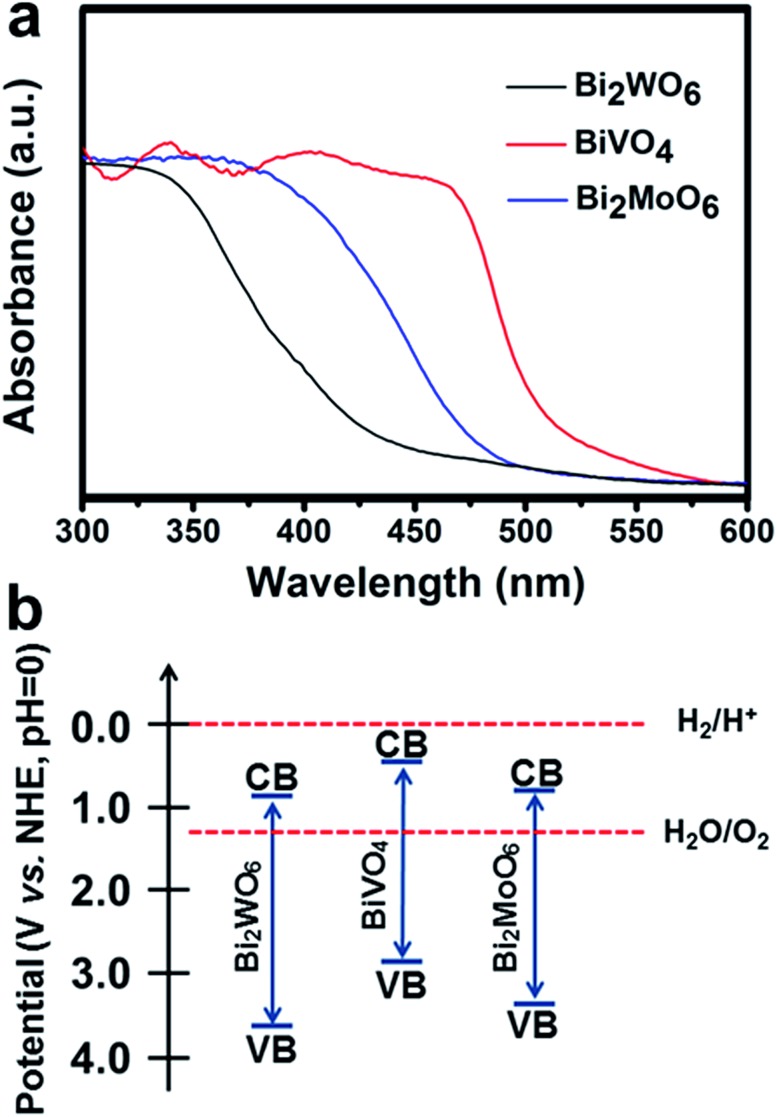
UV-Vis absorption spectra (a) and schematic band diagrams (b) of Bi_2_WO_6_, BiVO_4_ and Bi_2_MoO_6_.

The UV-Vis absorption spectra of the Bi_2_WO_6_, BiVO_4_, and Bi_2_MoO_6_ electrodes (Fig. 3a) show that the absorption edges of the three Bi-based photoelectrodes were 430 nm, 505 nm, and 480 nm. According to the Tauc plots (Fig. S3†), the band gaps (*E*_g_) for the Bi_2_WO_6_, BiVO_4_, and Bi_2_MoO_6_ porous nanoflakes were estimated to be 2.87, 2.46, and 2.52 eV, respectively, in accordance with a previous study about Bi-based binary metal oxides.^28^

Furthermore, the conduction band (CB) and valence band (VB) positions of the three Bi-based semiconductors at the point of zero charge can be calculated. The absolute electronegativity (*X*) of Bi_2_WO_6_, BiVO_4_, and Bi_2_MoO_6_ can be estimated to be 6.36, 6.04 and 6.31 from the literature.^30–32^ The band gap energy (*E*_g_) can be estimated from the Tauc plots shown in Fig. S3† and Table S4.† The conduction band edge of a semiconductor can be estimated using an equation as follows:^30^ *E*0VB = *X* – 4.5 + *E*_g_/2where 4.5 eV is the energy of free electrons on the hydrogen scale. The points of zero charge of the three samples (Bi_2_WO_6_, BiVO_4_, and Bi_2_MoO_6_) are 8.7, 3.0 and 6.1, respectively.^31,33,34^ Therefore, the calculated valence band edge at the point of zero charge can be corrected to pH 0 by the following method:*E*_VB_ = *E*0VB + pH_zpc_ × 0.059

Then, the valence band edge can be calculated using an equation as follows:*E*_CB_ = *E*_VB_ – *E*_g_

### 2.2 Reaction mechanism of the hydrothermal anion exchange

Upon the introduction of Na_2_WO_4_, NaVO_3_, and Na_2_MoO_4_ followed by hydrothermal treatments, the Bi_2_WO_6_, BiVO_4_, and Bi_2_MoO_6_ thin films were formed using the BiOI nanoflakes as templates, during which I^–^ ions were replaced by WO_4_^2–^, VO_3_^–^, and MoO_4_^2–^. The chemical equations of the anion exchange process can be shown as follows:2BiOI + Na_2_WO_4_ = Bi_2_WO_6_ + 2NaIBiOI + NaVO_3_ = BiVO_4_ + NaI2BiOI + Na_2_MoO_4_ = Bi_2_MoO_6_ + 2NaI

As the diffusion rates of the I^–^ ions and the WO_4_^2–^, VO_3_^–^, and MoO_4_^2–^ ions are unequal, a continuous ion exchange will lead to a porous morphology. To verify the binary hypothesis, an inductively coupled plasma optical emission spectroscopy (ICP-OES) study was employed. Taking Bi_2_WO_6_ as an example, a time-dependent ICP-OES study (Table S2†) shows the concentration of I^–^ ions in the residue after 0.5 h, 1 h, 2 h, and 3 h of hydrothermal anion exchange. As the anion exchange proceeds, the concentration of I^–^ ions increases at first and then reaches its maximum value after 2 h at 120 °C. In order to rule out the influence of I^–^ during the ICP-OES measurement, we put BiOI on FTO in the deionized water during the anion exchange and monitored the concentration of I^–^ ions in the residue after 0.5 h, 1 h, 2 h, and 3 h (Table S3†). The results indicate that the dissolution of BiOI can be ignored during the anion exchange process. Based on the morphology change from BiOI nanoflakes to Bi_2_WO_6_ porous nanoflakes, two effects of the BiOI template could be identified in the ion exchange process: (i) the Bi source for Bi_2_WO_6_ and (ii) the template for the nanoflake morphology.

To further explore the origin of the morphological change during the hydrothermal anion exchange process, FESEM images and XRD patterns of the Bi_2_WO_6_ porous nanoflakes were obtained. Fig. S4† shows the XRD patterns of the Bi_2_WO_6_ porous nanoflakes grown on FTO at 120 °C for 0.5, 1, and 2 h. From the XRD patterns, the peaks associated with the BiOI nanoflakes faded away with increasing exchange time, such as the peaks at 28.11° and 45.28°. On the contrary, the peaks associated with the Bi_2_WO_6_ porous nanoflakes gradually appeared with increasing growth time, such as the peaks at 31.61°, 46.88° and 55.68°. The morphology change during the reaction is shown in Fig. S5 (details in the ESI†). Continuous anion exchange is the reason for the morphological change.

### 2.3 PEC water oxidation performances of Bi_2_WO_6_, BiVO_4_, and Bi_2_MoO_6_

**Fig. 4:**
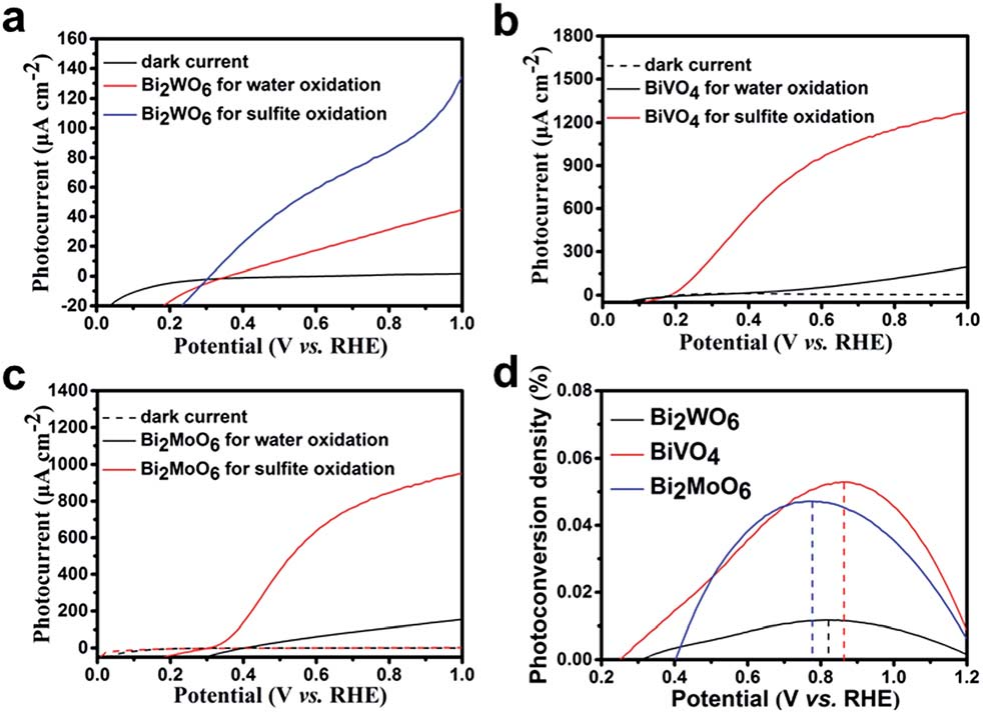
Current–potential plots for (a) Bi_2_WO_6_, (b) BiVO_4_ and (c) Bi_2_MoO_6_ under AM 1.5G (100 mW cm^–2^) illumination in a 0.1 M Na_2_SO_4_ aqueous electrolyte (pH = 6.8) and 1 M sodium sulfite (pH = 6.3); (d) photoconversion efficiency of the three samples for the water oxidation reaction.

The PEC performance of the Bi-based electrodes under AM 1.5G (100 mW cm^–2^) irradiation is examined (details in the ESI†). Fig. 4a shows the photocurrent density *vs.* potential (*I*–*V*) curves for the Bi_2_WO_6_ porous nanoflakes in a 0.1 M Na_2_SO_4_ aqueous electrolyte (pH = 6.8). The photocurrent of the Bi_2_WO_6_ porous nanoflakes is 41 μA cm^–2^ at a potential of 1.00 V *vs.* RHE, which is about four times larger than the values reported.^35,36^ When 1 M Na_2_SO_3_ was added to the electrolyte as a hole scavenger, a remarkable increase in the photocurrent of 130 μA cm^–2^ at 1.0 V *vs.* RHE was observed, with an onset of 0.30 V *vs.* RHE (Fig. 4a). It is observed that surface kinetics is an important factor for Bi_2_WO_6_ electrodes.

The PEC performances of BiVO_4_ and Bi_2_MoO_6_ were also measured under the same conditions as Bi_2_WO_6_. The photocurrent of the BiVO_4_ porous nanoflakes is 200 μA cm^–2^ at a potential of 1.00 V *vs.* RHE (Fig. 4b), while the samples synthesized by BiVO_4_ nanoparticles on FTO reached only 75 μA cm^–2^ at the same potential.^37,38^ For the sulfite oxidation reaction, the BiVO_4_ photoelectrodes show a considerably high value of 1280 μA cm^–2^ at 1.0 V *vs.* RHE, with an onset potential of 0.19 V *vs.* RHE. The porous nanoflake morphology of our BiVO_4_ photoelectrodes was completely different compared to other BiVO_4_ nanostructures. For photoelectrodes, an ordered morphology can increase the specific area and then provide more exposed reaction active sites. Compared with conventional preparation methods, our BiVO_4_ material has an ordered nanoflake morphology resulting from the anion exchange process.

Fig. 4c shows the photocurrent–potential plots of the Bi_2_MoO_6_ electrode. Compared with other reported Bi_2_MoO_6_ photoanodes, our Bi_2_MoO_6_ porous nanoflakes have better PEC performances.^18,34^ The maximum photocurrent of Bi_2_MoO_6_ photoanodes formed by the existing preparation methods was just about 13 μA cm^–2^ under visible light irradiation which limited the application of Bi_2_MoO_6_ photoanodes in PEC water oxidation reactions. The Bi_2_MoO_6_ porous nanoflakes exhibit outstanding photoactivities compared to other Bi_2_MoO_6_ photoanodes formed by the conventional preparation methods. The photocurrent values are still low compared to Choi's work.^27^ This may be because there was less photocatalyst per unit area and so the PEC performances were reduced. However, our preparation method was more convenient and easier to control. During the process of forming the BiVO_4_ electrode, we used NaVO_3_ solution as the precursor which was environmentally friendly instead of vanadyl acetylacetonate.

Additionally, we have acquired the photoconversion efficiencies of the three samples (details in the ESI†). The photoconversion efficiencies of Bi_2_WO_6_, BiVO_4_ and Bi_2_MoO_6_ were 0.0117% at 0.82 V *vs.* RHE, 0.0529% at 0.86 V *vs.* RHE and 0.0472% at 0.78 V *vs.* RHE (Fig. 4d) respectively. These values have a little increase compared with the references. Although such improvements haven't essentially changed the poor photoactivities of Bi-based electrodes, it can be seen that the answer is not out of reach.

**Fig. 5:**
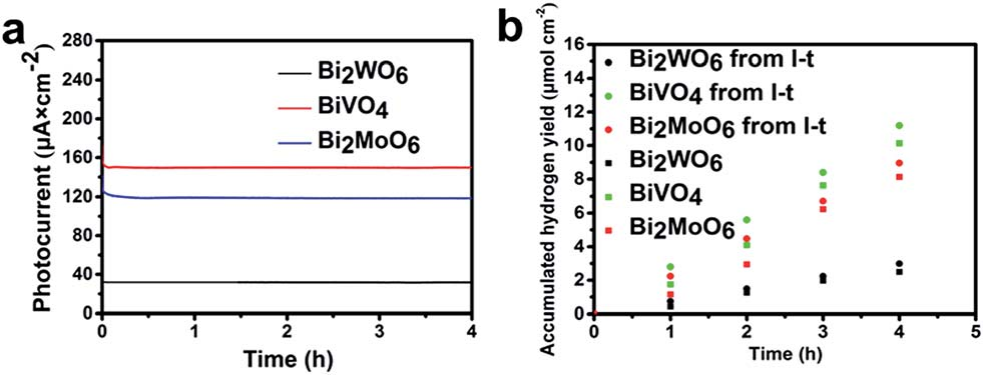
(a) Photocurrent–time plots, and (b) H_2_ production from water splitting reaction in a PEC cell of Bi_2_WO_6_, BiVO_4_ and Bi_2_MoO_6_ under AM 1.5G (100 mW cm^–2^) illumination in a 0.1 M Na_2_SO_4_ aqueous electrolyte (pH = 6.8). The constant potential of the photocurrent–time plots and H_2_ production in a PEC cell is 1.0 V (*vs.* RHE).

In order to explore the steady-state performance of the three Bi-based electrodes, the photocurrent–time (*I*–*t*) plots in a 0.1 M Na_2_SO_4_ aqueous electrolyte (pH = 6.8) under AM 1.5G irradiation at a constant applied bias of 1.0 V *vs.* RHE are shown in Fig. 5a. The *I*–*t* plots show that the photocurrents of Bi_2_WO_6_, BiVO_4_, and Bi_2_MoO_6_ photoanodes remain stable at 40, 150, and 120 μA cm^–2^, respectively, over a 4 h period. The good stability of semiconductors is an important precondition to be applied on a large scale. The *I*–*t* plots indicate that all of the three Bi-based electrodes have potential application in PEC water splitting reactions.

The hydrogen evolution rate of the three samples were also measured in a 0.1 M Na_2_SO_4_ aqueous electrolyte (pH = 6.8) under AM 1.5G at a constant applied bias of 1.00 V *vs.* RHE (Fig. 5b and details in the ESI†). The H_2_ production rates of all three samples remain constant with average values of 0.625, 2.533, and 2.031 μmol h^–1^ cm^–2^ during a 4 h reaction period. According to the photocurrent in Fig. 5a, the theoretical H_2_ production rates of all three samples (Bi_2_WO_6_, BiVO_4_ and Bi_2_MoO_6_) are 0.746, 2.798, and 2.238 μmol h^–1^ cm^–2^ and the faradaic efficiencies are 83.8%, 90.6%, and 90.8%, respectively. The loss of faradaic efficiency may be because of the heat loss in the process of reaction and the system error of our devices.

**Fig. 6:**
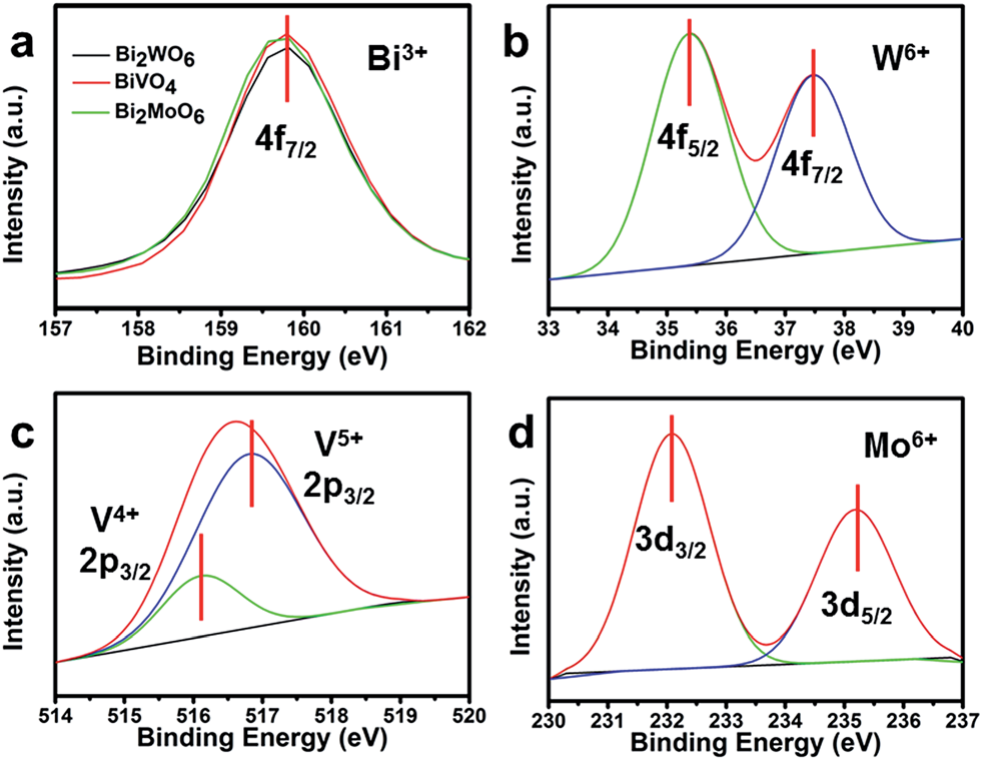
(a) Bi4f XPS spectra of the three Bi-based photoelectrodes; (b) W4f XPS spectra of Bi_2_WO_6_; (c) V2p XPS spectra of BiVO_4_; and (d) Mo3d XPS spectra of Bi_2_MoO_6_.

X-ray photoelectron spectroscopy (XPS) was employed to characterize the valence state of the elements in the three Bi-based semiconductors. The XPS peak at 159.8 eV for the three Bi-based samples corresponds to the Bi4f_7/2_ spectrum (Fig. 6a).^39,40^ For Bi_2_WO_6_, the characteristic peaks of W4f_7/2_ at 35.4 eV and W4f_5/2_ at 37.5 eV, are attributed to the W atoms in the 6+ oxidation state (Fig. 6b).^40^ Similar with Bi_2_WO_6_, the characteristic peaks of Mo3d_3/2_ and Mo3d_5/2_ can be observed in Fig. 6d.^41^ However, the asymmetric V2p_3/2_ signals were decomposed into two peaks at 516.1 and 516.8 eV for BiVO_4_ (Fig. 6c), attributable to the surface V^4+^ and V^5+^ species with V^5+^ : V^4+^ at about 79.8% : 20.2%.^42^ The surface V^4+^ defect sites may be a part of the slow surface kinetics because the unsaturated valence ions can be re-oxidized during the water oxidation process.^43^

## 3. Conclusions

In summary, we have demonstrated a simple and characteristic preparation method of hydrothermal anion exchange to synthesize Bi-based porous nanoflake electrodes. Starting from BiOI templates, a Bi_2_WO_6_ electrode can be easily prepared, which exhibits an ordered porous nanoflake morphology. Furthermore, the synthesis is based on an *in situ* growth method that can improve the crystallinity and the Bi-based electrodes have an ordered nanoflake morphology to increase the surface area. The BiVO_4_ porous nanoflakes on the FTO coated glass exhibit an improved photocurrent of 200 μA cm^–2^ for water oxidation and 1280 μA cm^–2^ for sulfite oxidation at a potential of 1.00 V *vs.* RHE under AM 1.5G irradiation. The Bi_2_WO_6_ and Bi_2_MoO_6_ electrodes also exhibit enhanced PEC performance compared with the electrodes formed by the conventional preparation methods. All three samples have excellent stability and display the potential to be promising photoelectrodes for hydrogen production. This general preparation method can successfully synthesize Bi-based photoanodes with nanoflake morphology. This method can simplify the synthesis process of Bi-based photoelectrodes and promote their applications in PEC water oxidation reactions.
