# Supplementary material for: Fabrication of porous nanoflake BiMOx (M = W, V, and Mo) photoanodes via hydrothermal anion exchange
Source: Chem Sci. 2016 Jun 24;7(10):6381–6. doi: 10.1039/c6sc01803c (PMC5356035; doi:10.1039/c6sc01803c)
Supplement: Supplementary file 1 [file SC-007-C6SC01803C-s001.pdf]

Supporting Information

Fabrication of Porous Nanoflake BiMOx (M=W, V, and Mo) Photoanodes via Hydrothermal Anion Exchange

Jijie Zhang, Tuo Wang, Xiaoxia Chang, Ang Li, and Jinlong Gong*

Key Laboratory for Green Chemical Technology of Ministry of Education, School of Chemical Engineering and Technology, Tianjin University, Collaborative Innovation Center of Chemical Science and Engineering, Tianjin 300072, China.

Corresponding author: jlgong@tju.edu.cn

Experimental section

Preparation of BiOI electrodes. A 0.04 M $Bi(NO_3)_3$ solution was prepared by dissolving $Bi(NO_3)_3 \cdot 5H_2O$ in 50 mL of a 0.4 M KI solution after its pH was adjusted to 1.7 by adding HNO_3. This Bi-based solution was added into 20 mL of absolute ethanol (100%) containing 0.23 M p-benzoquinone, and was vigorously stirred for 5 minutes. The electrodeposition process was achieved by a typical three-electrode cell. A fluorine-doped tin oxide (FTO) working electrode, a Ag/AgCl reference electrode, and a platinum counter electrode were used. Cathodic deposition was performed potentiostatically at -0.1 V *vs.* Ag/AgCl for 5 min.

Preparation of Bi_2WO_6, $BiVO_4$, and Bi_2MoO_6 electrodes. The Bi_2WO_6 electrodes were formed by a hydrothermal anion exchange process. Prior to the hydrothermal treatment, 0.1 M Na_2WO_6 solution was prepared by dissolving 1.32 g Na_2WO_6 in 40 mL deionized water. Subsequently, the BiOI film and prepared Na_2WO_6 solution was added to a Teflon-lined hydrothermal reactor and the hydrothermal reaction proceeds for 2 h at 120 °C. The reactor was then allowed to cool down to room temperature naturally and the resulting film was collected and washed with deionized water and dried in air. Finally, thermal treatment of the Bi2WO6 film was carried out at 500 °C for 2 h with a ramping rate of 2 °C min-1 in an air environment.

The $BiVO_4$ electrodes were formed by adding 40 mL of 0.2 M $NaVO_3$ and BiOI film into a Teflon-lined hydrothermal reactor and the hydrothermal reaction proceeds for 2 h at 160 °C. Thermal treatment of the $BiVO_4$ film was carried out at 450 °C for 2 h with a ramping rate of 2 °C min^{-1} in an air environment.

In order to prepare Bi_2MoO_6 electrodes, 40 mL of the mixed solution which contains 0.4 M Na_2MoO_4, 20 mL deionized water and 20 mL ethyl alcohol was adjusted the pH to 5 with adding hydrochloric acid. Then the as-prepared Na_2MoO_4 solution and BiOI film were added into a Teflon-lined hydrothermal reactor and the hydrothermal reaction proceeds for 6 h at 160 °C. Thermal treatment of the Bi_2MoO_6 film was carried out at 400 °C for 2 h with a ramping rate of 2 °C min-1 in an air environment.

Structural and Optical Characterization. X-ray diffraction patterns were recorded with a Bruker D8 Focus operating at 40 kV and 40 mA equipped with a nickel-filtered Cu Kα radiation (λ = 1.54056 Å) and operating in a 2θ range of 20 – 80 ° at a scan rate of 6 per minute. The morphologies were characterized by field emission scanning electron microscope (FESEM, S-4800). Elemental analysis was carried out by energy dispersive spectroscopy (EDS) at 15 kV. UV–visible reflectance spectra and transmittance spectra of Bi-based electrode were obtained on a SHIMADZU UV-2550 spectrophotometer. The iodine content was determined by inductively coupled plasma optical emission spectroscopy (ICP-OES) (Vista-MPX, Varian). The specific surface areas were calculated from the isotherms using the BET method.

PEC Measurements

PEC measurements were performed in 0.1 M Na_2SO_4 (pH 6.8) using a standard quartz 3-electrode cell with Pt foil as the counter electrode, Ag/AgCl as the reference electrode and the Bi_2WO_6, $BiVO_4$, and Bi_2MoO_6 as the working electrode. In order to simulate sunlight, a 300 W xenon lamp (PE300BUV, CERMAX) equipped with an AM 1.5 filter was used as the light source, and the

power intensity of the light was calibrated to 100 mW/cm^2. An electrochemical workstation (IVIUM CompactStat.e20250) was used to measure the current-voltage (I-V) characteristic of the electrode, with a scan rate of 50 mV s^{-1}. We also measure the I-V curves of the three samples under visible light irradiation with wavelength ≥420 nm (Fig. S5).

Calculation of the amount of hydrogen evolution

To quantitatively determine the amount of H_2 produced from the reduction of water, a three-electrode system was employed. An aqueous Na_2SO_4 (0.1 M, pH 6.8) was used as the electrolyte solution. The sample was irradiated under irradiation of air mass 1.5 global (AM 1.5G) full sunlight (100 mW cm^{-2}). The gas collected from the platinum counter electrode was analyzed by an online gas chromatograph (GC2060, Shanghai RuiMin Electronics Group) with a thermal conductivity detector (TCD) using He as the carrier gas. For this purpose, a customer-designed air-tight PEC cell was used and the amount of H_2 was measured every 1 h at a constant bias of 0.4 V *vs.* Ag/AgCl under AM 1.5G illumination.

Calculation of the band gap of Bi_2WO_6, $BiVO_4$, and Bi_2MoO_6 electrodes

The optical bandgap of three samples could be calculated according to the equation:

$$\alpha hv=A(hv-Eg)^n$$

where h is Planck's constant, v is the frequency of light, A is a material related constant, Eg is bandgap energy, and n equals to 2 for an indirect semiconductor or 0.5 for a direct semiconductor. $BiVO_4$ is a direct semiconductor and Bi_2WO_6 and Bi_2MoO_6 are indirect semiconductors.

Calculation of the solar to hydrogen (STH) efficiencies

We have acquired the optimal photoconversion efficiency of the three Bi-based samples according to the equation:

$$\eta=I(1.23-V)/P_{light}$$

Where I is photocurrent density at the measurement applied bias, V is the applied bias (*vs.* RHE), and P_{light} is the incident light intensity of 100 mW cm^{-2} (AM 1.5G). In 0.1 M Na_2SO_4 electrolyte, the reversible hydrogen electrode (RHE) potential can be converted from the Ag/AgCl reference electrode as:

$E_{RHE} = E_{Ag/AgCl}+0.059\times pH+ E^o_{Ag/AgCl}$, where $E^o_{Ag/AgCl} = 0.197$ V at 25 ºC

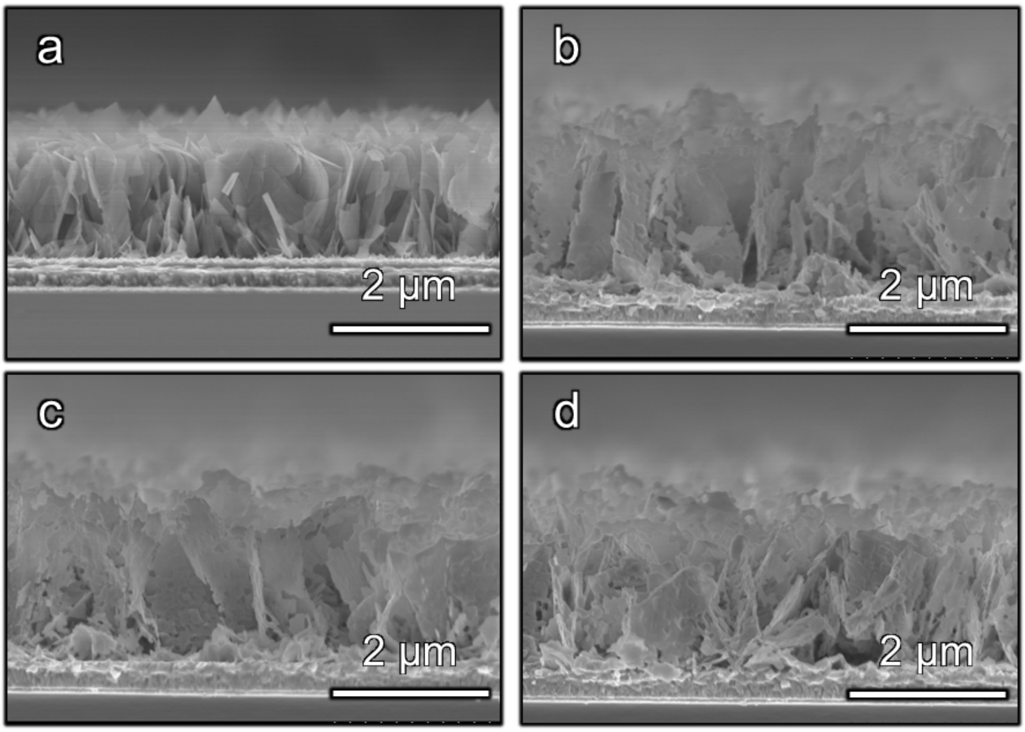

Fig. S1. Cross-sectional FESEM images of (a) BiOI, (b) Bi_2WO_6, (c) $BiVO_4$, and (d) Bi_2MoO_6.

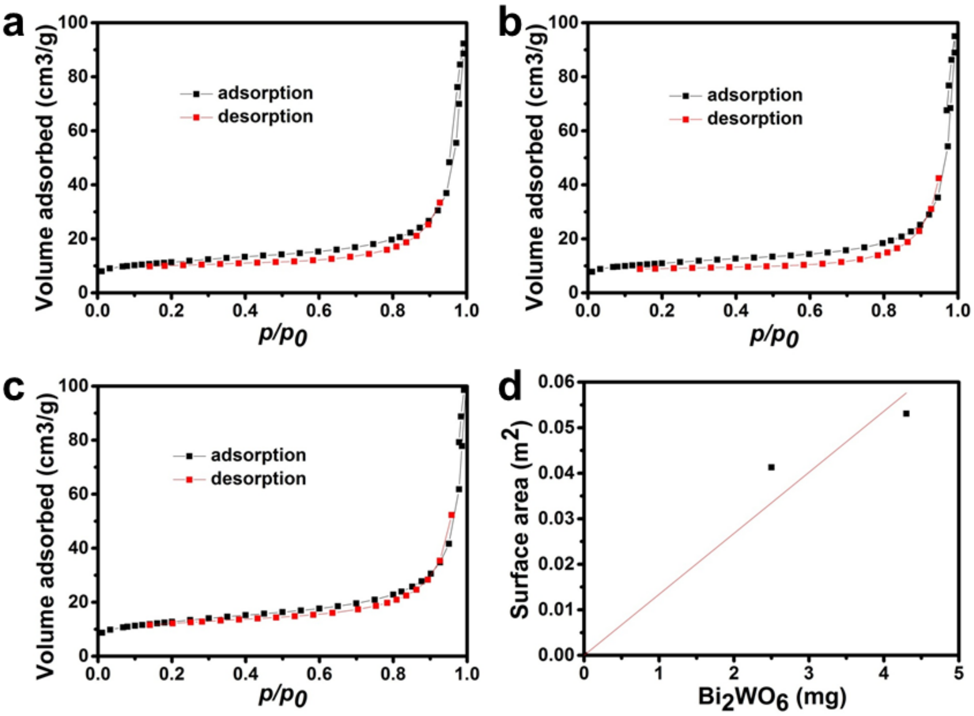

Fig. S2. Nitrogen adsorption isotherms of (a) 50 mg P-25, (b) 2.5 mg Bi_2WO_6 + 50 mg P-25 and (c) 4.3 mg Bi_2WO_6 + 50 mg P-25. (d) The fitting line of Bi_2WO_6 weight and surface area.

Considering the quality of the sample, we measure the specific surface area by the Brunauer-Emmett-Teller (BET) Method and use P-25 nanoparticles as the internal standard. Taking Bi_2WO_6 as an example, the surface areas of three samples, which contain different weight of Bi_2WO_6 (0 mg, 2 mg and 4 mg) and 50 mg P-25 nanoparticles, were measured. The surface area of Bi_2WO_6 can be extracted by subtracting the surface area of P-25 nanoparticles (Fig. S2). The specific surface area of three Bi-based photoelectrodes can be estimated about 13.4 m^2/g, 27.3 m^2/g and 30.0 m^2/g, respectively.

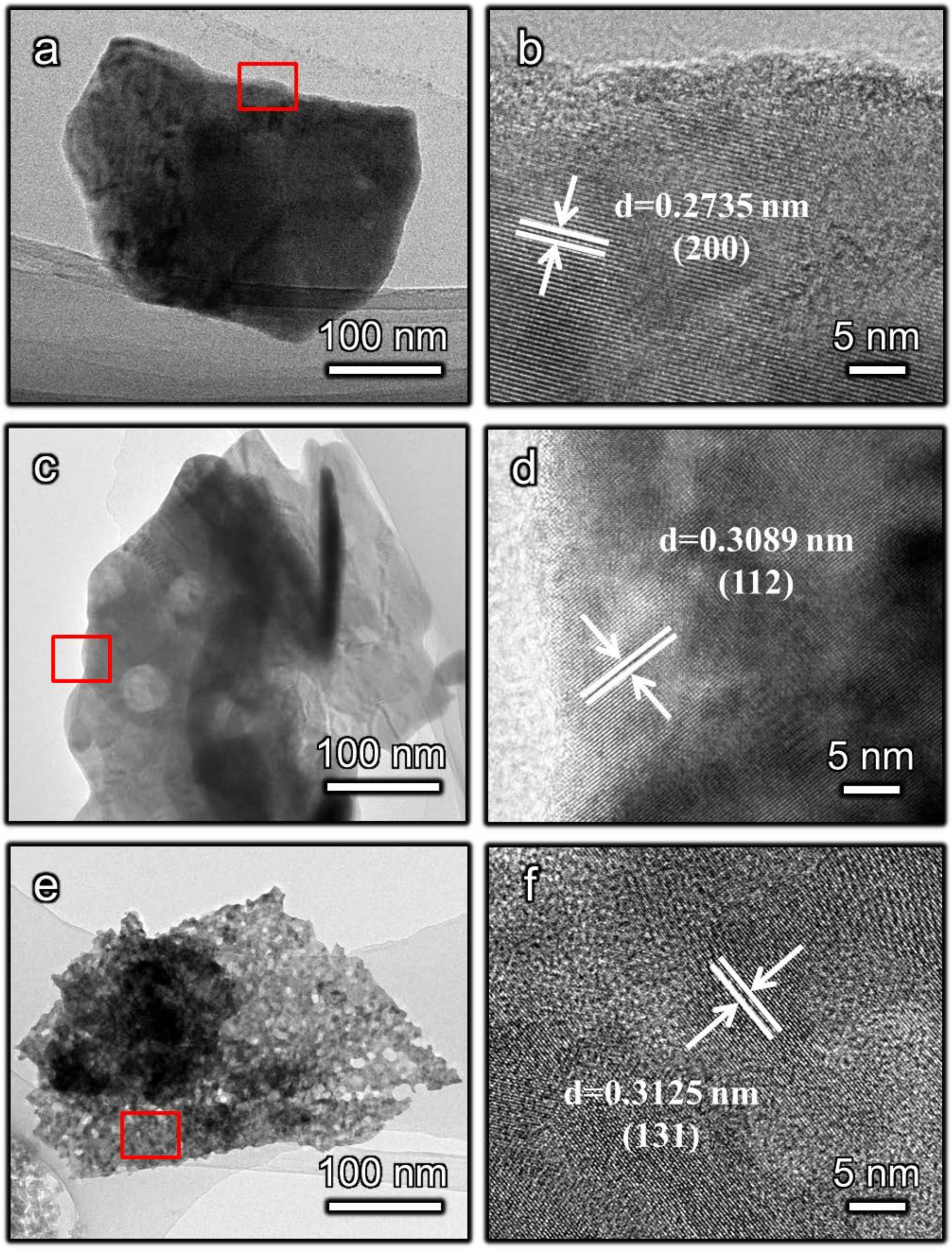

Fig. S2. Low-magnification TEM image and HRTEM image of Bi_2WO_6 (a and b), $BiVO_4$ (c and d), and Bi_2MoO_6 (e and f).

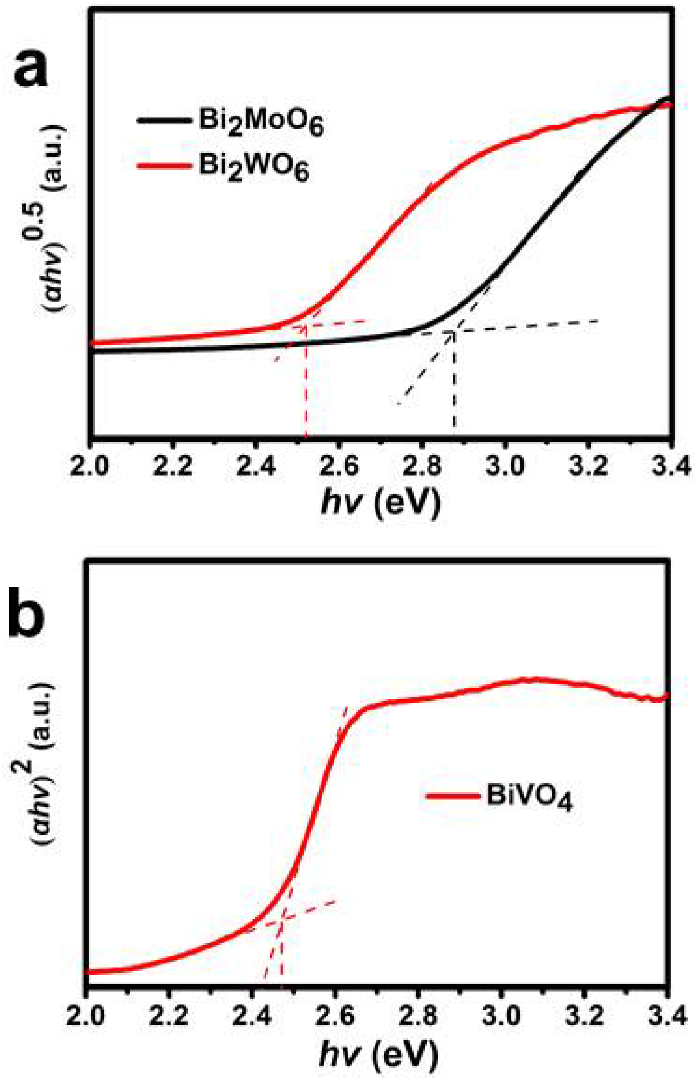

Fig. S3. Tauc plots of (a) Bi_2WO_6, Bi_2MoO_6, and (b) $BiVO_4$.

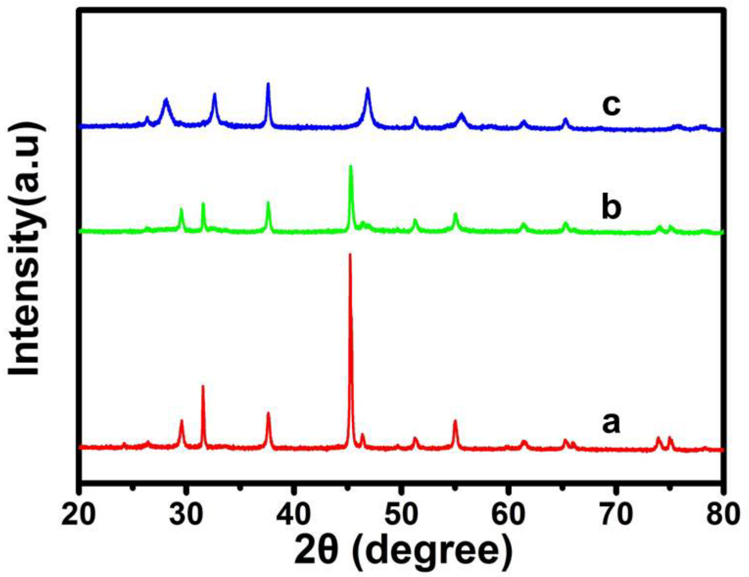

Fig. S4. XRD patterns of Bi_2WO_6 porous nanoflakes grown on FTO at 120 °C for (a) 0.5, (b) 1, and (c) 2 h.

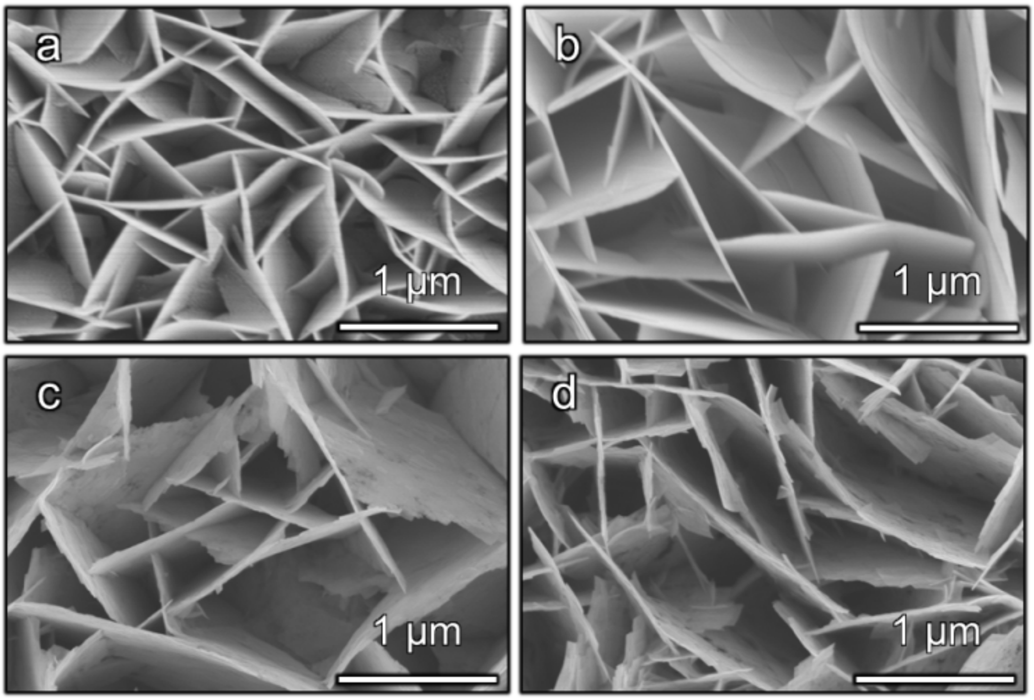

Fig. S5. FESEM images of Bi_2WO_6 porous nanoflakes grown on FTO at 120 °C for (a) 0, (b) 0.5, (c) 1, and (d) 2 h.

Fig. S5 shows the SEM images of Bi_2WO_6 porous nanoflakes grown on FTO at 120 °C for 0.5, 1, and 2 h. When the hydrothermal anion exchange time was shorter than 0.5 h at 120 °C, no obvious porous structure was formed on the surface of nanoflake templates (Fig. S5a and b). When the hydrothermal anion exchange time increased to 1 h, there are irregular nanoporous nanostructure on the surface on BiOI nanoflakes and meanwhile the edges of the nanoflakes become rough (Fig. S5c). As the hydrothermal anion exchange time reaches 2 h, the porous nanoflake morphology is completely developed (Fig. S5d). In the formation process of nanoporous morphology, the different diffusion rates of I^- and WO_4^{2-} act the leading role In the process of formation, which is known as the Kirkendall effect.[1, 2]

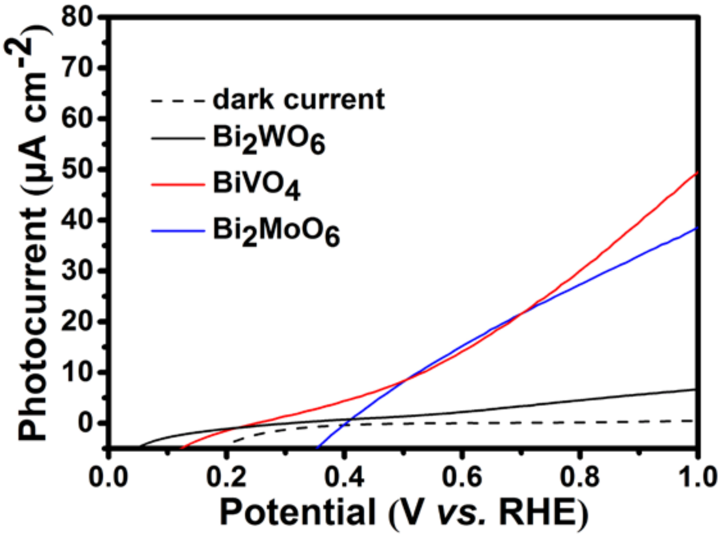

Fig. S6. Current-potential plots for Bi_2WO_6, $BiVO_4$ and Bi_2MoO_6 under visible light illumination with wavelength ≥420 nm in a 0.1 M Na_2SO_4 aqueous electrolyte (pH 6.8).

Table S1. Average atomic ratios (metal basis) in the Bi_2WO_6, $BiVO_4$, and Bi_2MoO_6 electrodes obtained by EDS analysis.

	Bi_2WO_6	$BiVO_4$	Bi_2MoO_6
Average ratio	Bi:W=2.16:1	Bi:V=0.96:1	Bi:Mo=2.07:1

Table S2. The concentration of I^- ions in the residue after 0.5 h, 1 h, 2 h, and 3 h hydrothermal anion exchange measured by ICP-OES.

	0 h	0.5 h	1 h	2 h	3 h
Concentration of I^- (ppm)	0.000	33.665	59.287	121.481	121.793

Table S3. The concentration of I^- ions in the residue after 0.5 h, 1 h, 2 h, and 3 h heating in the deionized water measured by ICP-OES.

	0 h	0.5 h	1 h	2 h	3 h
Concentration of I^- (ppm)	0.000	0.122	0.202	0.235	0.334

Table S4. Absolute electronegativity, band gap energy, energy levels of calculated conduction band edge and valence band edge for Bi_2WO_6, $BiVO_4$, and Bi_2MoO_6.

Semiconductor	Absolute electronegativity (X)	Band gap energy (eV)	Conduction band edge (V *vs.* NHE)		Valence band edge (V *vs.* NHE)	
			pH_{zpc}	pH=0	pH_{zpc}	pH=0
Bi_2WO_6	6.36	2.87	0.42	0.94	3.3	3.81
$BiVO_4$	6.035	2.46	0.31	0.49	2.77	2.95
Bi_2MoO_6	6.31	2.52	0.55	0.91	3.07	3.43

References

1. X. Zhao, Y. Wu, W. Yao and Y. Zhu, *Thin Solid Films*, 2007, **515**, 4753-4757.
2. H. Yu, Z. Zhu, J. Zhou, J. Wang, J. Li and Y. Zhang, *Appl. Surf. Sci.*, 2013, **265**, 424-430.
